# Empowering African genomics for infectious disease control

**DOI:** 10.1186/s13059-014-0515-y

**Published:** 2014-11-07

**Authors:** Onikepe A Folarin, Anise N Happi, Christian T Happi

**Affiliations:** Department of Biological Sciences, College of Natural Sciences, Redeemer’s University, P.M.B. 230, Ede, Osun State Nigeria; African Center of Excellence for Genomics of Infectious Diseases (ACEGID), Redeemer’s University, P.M.B. 230, Ede, Osun State Nigeria; Department of Veterinary Pathology, Faculty of Veterinary Medicine, University of Ibadan, Ibadan, Nigeria

## Abstract

At present, African scientists can only participate minimally in the genomics revolution that is transforming the understanding, surveillance and clinical treatment of infectious diseases. We discuss new initiatives to equip African scientists with knowledge of cutting-edge genomics tools, and build a sustainable critical mass of well-trained African infectious diseases genomics scientists.

## Background

The negative economic and social impact of infectious diseases in Africa cannot be overemphasized; they are leading causes of death and economic losses in the continent. One of the major challenges in the control of infectious diseases in Africa is inadequate knowledge and understanding of the pathogens and their various hosts. However, as genomic technologies have become available, and the sequencing of both the human genome and the genomes of many pathogens has been completed, we are witnessing a revolution in the way infectious disease research is approached and conducted. This progress has also brought about enormous health, scientific and economic benefits [1].

The availability of sequencing data from infectious pathogens represents a unique opportunity for the identification of new drug and vaccine targets, which potentially have value for disease management and control. These data have, however, been predominantly benefiting researchers, institutions and laboratories in North America, Australia, Europe and Asia, contributing to the increasing economic, scientific and genomics knowledge gap between these geographic regions and Africa. This is partly due to the fact that while governments in countries such as the US and UK have increased their investment in genomics research, there is a paucity of governmental, regional political or economic organizational funding in Africa for genomicists to address the burden of infectious diseases. Despite the technological advances and significant reductions in the cost of genomic research, African scientists are yet to use genomics-based knowledge and tools to provide novel insights into disease etiology, diagnosis, and therapy for some of the most intractable and devastating diseases on the continent, including malaria, HIV-AIDS and tuberculosis. If the dearth of genomics research involving Africans persists, the potential health and economic benefits emanating from genomics may elude the entire continent [2].

There is therefore an important and urgent need to facilitate the establishment of a vibrant research and academic environment that is free of outside influences, that transcends national boundaries, and that ensures the conduct of relevant, responsive, ethical and high-quality translational genomics-based research on infectious diseases in Africa. This will depend, in part, on the ability of African scientists to: 1) acquire the expertise and facilities necessary to lead high-quality genomics-based research aimed at understanding infectious diseases relevant to African populations; and 2) become internationally competitive in genomics science and its applications.

## New initiatives for the genomics of infectious diseases in Africa

Some new initiatives have recently been put in place to empower African researchers to overcome the challenges described above and to unlock the potential for infectious diseases control through genomics-based approaches. These are the H3Africa consortium (www.h3africa.org), which is funded by the National Institutes of Health (NIH) and the Wellcome Trust, and the African Center of Excellence for Genomics of Infectious Diseases-ACEGID (www.acegid.org), which is funded by the World Bank. Both initiatives are focused on capacity building, as well as on specific scientific goals.

The H3Africa initiative focuses on both non-communicable and infectious diseases, and has a major objective to award research grants directly to African institutions in which principal investigators are based. This allows African scientists to develop and direct their independent research agendas. The program also encourages the formation of intra-continental collaborations, and the development of specific infrastructural elements, such as African-based bio-repositories and a pan-African bioinformatics network (H3ABio-Net). Furthermore, the H3Africa initiative also includes training programs aimed at retaining African scientists on the continent to help build a sustainable critical mass of genomics-based researchers [2].

The World-Bank-funded ACEGID program, which is based at Redeemer’s University, Mowe, Nigeria, focuses strictly on infectious diseases. ACEGID’s research-related goals are focused on characterizing fevers of unknown origins through microbial metagenomics. It uses field-deployed and state-of-the-art genomics technology to identify the pathogens driving febrile illness. ACEGID also aims to create a foundation for African scientists to carry out tractable and important genetic research projects entirely in their own country. At present, clinical diagnostics are unavailable for many of the pathogens that cause febrile illnesses across the sub-continent. In light of this gap, the project also seeks to employ new sequencing technologies and bioinformatics to discover novel pathogens, as well as to develop and deploy new tools for field diagnosis of new and known microorganisms.

By building human and infrastructural capacity for state-of-the-art genome sequencing and field-deployable genetics tools to inform work on microbial infection in Africa, the ACEGID program can support the clinical management of the most devastating diseases, and can enable a surveillance network for some of the world’s greatest health threats. This has been well illustrated by the current Ebola epidemic in the West African regions. ACEGID-established laboratories and researchers performed the first diagnosis of Ebola virus disease in Nigeria and Sierra Leone and were able to track its origin and evolution in West Africa [3].

ACEGID plans to establish lasting scientific capacity in genomics through complementary research and education-based goals related to the detection and control of infectious diseases. The educational goals of ACEGID are to: 1) develop a critical mass of well-trained African genomics scientists; 2) empower African researchers to utilize genomics-based tools towards the control and elimination of infectious diseases; 3) create genomics curricula to support and promote cutting-edge genomics-based research; and 4) engage communities in prevention efforts and public health education.

Despite the apparent success of H3Africa and ACEGID, one of the major challenges to building critical mass for genomic research in Africa will be the ability of African institutions to retain scientific leaders who are capable of developing and maintaining sustainable research programs. This can only be achieved through the support of national governments and of regional political and economic organizations in providing sustained funding for all research fields, including infectious diseases genomics and research infrastructure development. In addition, African countries should be building their infrastructures to promote local innovation, and to retain the value of their human and microbial genetic diversity. There is also a need to develop and foster north–south, south-south (intra- and inter-African institution) collaborations to develop the necessary infrastructure and biomedical research culture required to promote genomics-based research for infectious diseases control in Africa.

## Conclusions

New initiatives to empower African scientists in genomics have the potential not only to reduce the ‘genomics divide’ between Africa and the rest of the World, but also to change positively the infectious diseases research and academic landscapes on the continent. The initial success of the H3Africa and ACEGID initiatives has shown that well thought through and articulated programs can enable African scientists to pursue high-impact projects. This work will not only bring regional academic and research success for African science, but will also contribute to the improvement of global public health.

## Abbreviations

ACEGID: African Center of Excellence for Genomics of Infectious Diseases
H3Africa: Human Hereditary and Health in Africa
NIH: National Institute of Health

## References

[1] Lander ES (2011). Initial impact of the sequencing of the human genome. Nature.

[2] H3Africa Consortium (2014). Enabling African scientists for the genomic revolution. Science.

[3] Gire SK, Goba A, Andersen KG, Sealfon RS, Park DJ, Kanneh L, Jalloh S, Momoh M, Fullah M, Dudas G, Wohl S, Moses LM, Yozwiak NL, Winnicki S, Matranga CB, Malboeuf CM, Qu J, Gladden AD, Schaffner SF, Yang X, Jiang PP, Nekoui M, Colubri A, Coomber MR, Fonnie M, Moigboi A, Gbakie M, Kamara FK, Tucker V, Konuwa E (2014). Genomic surveillance elucidates Ebola virus origin and transmission during the 2014 outbreak. Science.

